# Building a health system resilience framework: national, state, regional, and local perspectives

**DOI:** 10.1016/j.lana.2025.101334

**Published:** 2025-12-11

**Authors:** Marco Antonio Catussi Paschoalotto, Eduardo Alves Lazzari, Rudi Rocha, Adriano Massuda, Marcia C. Castro

**Affiliations:** a Research Center in Political Science, School of Economics, Management and Political Science, University of Minho, Braga, Portugal; b David Rockefeller Center for Latin American Studies (DRCLAS), Harvard University, Boston, MA, USA; c Sao Paulo School of Business Administration (FGV EAESP), Fundação Getúlio Vargas, Sao Paulo, Brazil; d Institute for Health Policy Studies (IEPS), Brazil; e Department of Global Health and Population, Harvard T H Chan School of Public Health, Boston, MA, USA

**Keywords:** Health system resilience, Decentralized, Health system, Unified health system, Governance

## Abstract

Health system resilience (HSR) is essential to sustaining equitable essential functions under acute and chronic stressors in decentralized systems. We developed and validated a Brazil-tailored HSR framework that distinguishes steady-state performance from resilience-specific capacities and assigns responsibilities across federal, state, regional, and municipal levels. Using a three-phase qualitative deductive–inductive approach with 48 international and national experts, we identified nine dimensions, 18 subdimensions, and 65 indicators that prioritise governance coherence, surge workforce strategies, emergency regulation, real-time monitoring, and access to critical technologies. The framework clarifies boundaries between general health system performance and adaptive, absorptive, and transformative functions, and specifies how managers can apply it in practice through structured scoping, mapping, scoring, prioritisation, planning, and monitoring steps. Although designed for Brazil's Unified Health System (SUS), the development logic generalises to other decentralised contexts with appropriate re-allocation of responsibilities and calibration to national financing rules. This policy-facing tool supports actionable resilience strengthening in complex, multi-level systems.

## Introduction

Since the early 2000s, frameworks to assess health systems have been central to global health discussions.^1^ Murray and Frenk's seminal work in 2000 laid the foundation for structured health system assessments,^2^ later consolidated into the “building blocks” framework by the World Health Organization (WHO) in 2010.^3^ These efforts emphasized the critical components of health systems, setting a global benchmark for health system evaluation. However, the emergence of new global crises has underscored the need for a deeper focus on health system resilience (HSR), prompting significant academic and policy attention in recent years.^4^^,^^5^

The HSR concept gained traction following economic crises and public health emergencies, such as the Ebola epidemic in West Africa.^6^ The first HSR frameworks emerged to address these challenges. In 2013, Thomas and colleagues developed frameworks to examine the impact of economic downturns,^7^ while in 2015, Kruk and colleagues focused on HSR during the Ebola outbreak.^8^ Also, international organizations developed studies on HSR and tried to improve the knowledge acquired in different outbreaks.^9^, ^10^, ^11^, ^12^ Subsequent studies broadly expanded the resilience lens to encompass public health emergencies.^13^^,^^14^ Before the COVID-19 pandemic, HSR studies continued to grow. They focused on three areas: a) to define the HSR concept,^15^, ^16^, ^17^, ^18^ b) to propose HSR capacities, capabilities or frameworks,^19^^,^^20^ and c) to analyze new country experiences of resilient health systems.^21^, ^22^, ^23^ We define health system resilience as the capacity to absorb, adapt, and transform while sustaining equitable essential functions under acute and chronic stressors.^6^^,^^24^^,^^25^ Shocks include not only epidemics and disasters, but also ongoing fiscal crises, governance volatility, and entrenched inequities.^22^^,^^26^^,^^27^

Therefore, resilience interacts with foundational system attributes.^28^ Comparative literature shows that universal, publicly financed systems tend to achieve more equitable outcomes than fragmented, market-oriented models.^29^ However, these baseline capacities do not substitute for the adaptive, absorptive, and transformative functions required under shocks.^30^^,^^31^

No previous context influenced the HSR research field as the COVID-19 pandemic did.^4^^,^^6^ It catalyzed a surge in HSR research, with studies (a) exploring an understanding of the preparedness mechanisms used by health systems and governments^32^, ^33^, ^34^; (b) describing health system responses to the COVID-19 pandemic^25^^,^^35^^,^^36^; and (c) analyzing the recovery and learning strategies to think about the future of HSR.^37^, ^38^, ^39^ More recently, a handbook on health system resilience has been launched with worldwide experiences and organizing the knowledge acquired in different contexts.^40^

Several HSR studies were also undertaken by international organisations and non-governmental initiatives during the COVID-19 pandemic.^6^^,^^41^ Right before and at the beginning of the COVID-19 pandemic, the Global Health Security (GHS) Index was a reference framework,^42^ but the pandemic revealed its limitations.^43^^,^^44^ The WHO Europe and the European Observatory on Health Systems and Policies advanced the development of dimensions and indicators to measure HSR.^24^^,^^45^, ^46^, ^47^, ^48^ Initiatives like the Partnership for Health System Sustainability and Resilience (PHSSR) further reflect ongoing efforts to contextualize resilience across different health systems.^49^^,^^50^

Despite these advancements, existing frameworks often generalize resilience across varying health system contexts, limiting their applicability to specific realities.^41^^,^^51^, ^52^, ^53^ For example, in a country like Brazil, highly complex across multiple socio-political dimensions, a tailored HSR framework is essential.^54^^,^^55^ These attributes demand a resilience framework that captures these nuances and addresses the health system's ability to withstand and adapt to shocks while maintaining equity and service delivery.

In this paper, we propose a comprehensive resilience framework for the Brazilian health system (*SUS—Sistema Único de Saúde*), the first to be developed specifically for one country, for different government levels and with dimensions, subdimensions and specific indicators. Moreover, we also propose to deepen the HSR field by (a) discussing the differences between the health system and HSR indicators, (b) dividing the HSR indicators by different government levels, and (c) showing the advancements in an HSR framework.

Developing a context-sensitive resilience framework for Brazil allows us to understand health system dynamics in fragmented settings better. Using a unique set of 48 international and national health system experts and capturing the country's specific challenges and strengths, our framework can inform targeted policy interventions, ultimately contributing to the HSR literature and designing more resilient and equitable health systems. Most frameworks do not allocate indicators across governance levels in decentralised systems or explicitly distinguish system performance from resilience capacities. We address these gaps by developing and validating a multilevel, indicator-based framework.

## Methods

We applied a qualitative deductive-inductive approach divided into three phases: phase 1–create the HSR dimensions from an international perspective; phase 2–adapt the dimensions to the Brazilian health system and establish the indicators of each dimension; and phase 3–validate the Brazilian HSR framework through experts' agreement. We choose the deductive-inductive approach to (a) identify already existent health system resilience dimensions, subdimensions and indicators—deductive; and (b) discover new dimensions and indicators from the health system resilience experts—inductive.^23^^,^^56^

### Context—Unified Health System (SUS)

Brazil's Unified Health System (SUS) is one of the largest public health systems in the world, providing universal, free, and comprehensive care to the country.^21^ Established by the 1988 Constitution, the SUS operates on principles of universality, equity, and integrality, ensuring access to a wide range of services, from primary care to specialized and hospital services. The system is funded predominantly through social contributions and managed through a decentralized governance structure involving federal, state, and municipal governments.^50^

The SUS is characterized by its vast scope, including programs for immunization, maternal and child health, chronic disease management, and infectious disease control. Primary care plays a pivotal role, with the Family Health Strategy (FHS) as a cornerstone, delivering community-based, preventive, and curative services.^21^ Despite its achievements, the SUS faces significant challenges, including regional disparities, fragmented care delivery, and resource constraints.^35^^,^^57^ The SUS offers an essential health safety net for the Brazilian population and has demonstrated resilience in responding to public health crises, such as the COVID-19 pandemic.^58^ Even under mismanagement during the COVID-19 pandemic, the SUS remained responsive during the crisis through its inter-federative governance mechanisms and health workforce response.^54^^,^^59^

Therefore, SUS operates amid recurrent political volatility, persistent fiscal constraints, and marked socioeconomic and regional inequalities in access and capacity.^21^^,^^60^ These everyday stressors interact with acute events and require resilience strategies that prioritise continuity of essential public health functions across territories.^61^^,^^62^

### Data collection and analysis

**Fig. 1:**
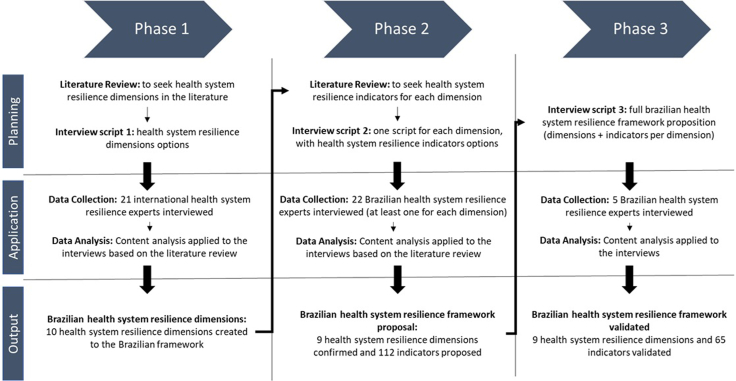
**Data collection and analysis phases**.

The data collection and analysis followed the three phases represented in Fig. 1. Phase 1 is dedicated to foundational planning and framework development. It focuses on identifying key dimensions of health system resilience, drawing insights from literature reviews (Appendix 1) and then crossing with the 21 experts' interviews. The phase 1 interviews were anchored in the WHO Building Blocks^3^ to ensure comprehensiveness and comparability with established performance domains; we then iteratively translated inputs into resilience-specific subdimensions and indicators, separating steady-state performance from adaptive capacities. Initial drafts of dimensions are crafted during this phase by a deductive-inductive approach, forming ten dimensions, which are the backbone of the framework.

Phase 2 amplifies the initial framework by testing the dimensions and developing the indicators to represent each. Based on phase 1 and the new literature review to collect more indicators for each one of the dimensions, 22 Brazilian experts were interviewed (Appendix 2), generating through deductive-inductive analysis the Brazilian HSR framework proposal. Finally, the final phase validates the framework by interviewing five experts and defining the government and geographical level of the indicators using a deductive approach.

### Search strategy and selection criteria

We searched PubMed, Scopus, Web of Science, WHO/PAHO repositories, and grey literature (from 2010 to 2023) using MeSH and free-text terms (“health system resilience”, “framework”, “indicator∗”, “governance”, “decentralised”, “Brazil”, “index”, “building blocks”, “CoReS”, and “roadmap”). Reference lists were hand-searched. The inclusion criterias were being a conceptual/measurement work on HSR or related indices, and Brazil-specific applications. The exclusion criteria were being purely clinical resilience or single-facility studies without system implications. This structured, non-systematic review, informed phase 1 and 2 deductive domains.

### Rigour and reporting

The interviews experts were selected purposively, based on their academic and/or practical knowledge and expertise in the health system resilience dimensions and indicators. The experts were contacted by email, signed a consent form and the interviews were realized and recorded through the zoom platform.

The reporting process followed the COREQ elements (team/reflexivity; study design; analysis),^63^ available in Appendix 4. We used triangulation across three phases, prespecified codebooks, dual-reviewed coding in phase 2 subsamples, and audit trails. A Delphi was considered, but we prioritised semi-structured interviews to elicit context-specific insights across governance levels and then used a structured validation interview (Phase 3) to confirm indicator inclusion and level allocation. Interview guide and codebook are provided in Supplementary Material, Appendix 1 and 2.

## Results

Our qualitative deductive-inductive approach included a unique sample of experts with diverse characteristics, covering multiple geographies and institutional settings (Table 1). Specifically, the interviews include experts from all continents in the world, the Brazilian experts (phases 2 and 3) have experience in all three government levels across different regions of the country, phase 2 included at least one expert of each dimension established in phase 1, and all the experts interviewed in phase 3 worked in the health department of the three government levels. All interviews of the three phases happened between November 5th 2021, and September 19th 2023, with an average duration of 49 min.

**Table 1:** HSR Framework summarized results.

Dimension	Sub-dimension	Number of indicators (Phase)	
		Confirmed/Proposed (One and Two)	Confirmed (Three)
Governance	Political and Administrative Structure	7	4
	Stakeholders and Institutional Support	6	3
Leadership	Background	7	3
	Leadership Capacity	4	2
Regulation (care coordination across the health system)	Regulation	2	2
	Structure and Healthcare Network	6	4
	Care Management	5	4
Financing	Financial Resources	7	6
	Management Tools	5	3
Health workforce	Distribution of Professionals	5	5
	Management Tools	8	5
Physical resources	Management Tools	5	5
	Care Network	4	1
	Infrastructure	5	3
Medicines	Management Tools	4	3
	Regulatory Market	7	0
	Pharmaceutical Services	2	2
Technology	Information Systems	7	3
	Communication and Support	3	2
Service delivery	Public Health	5	2
	Primary Care	4	0
	Specialized Care	4	3
**9**	**18**	**112**	**65**

Note: the subdimensions regulatory market and primary care did not have any indicators confirmed in phase 3, summing 18 subdimensions in the final.

Phases 1 and 2 of our qualitative deductive-inductive approach generated a Brazilian HSR framework proposal with 9 dimensions and 112 indicators (Supplementary Material, Appendix 3). The final framework has nine health system resilience dimensions, 18 subdimensions and 65 indicators validated at national, state, regional and local levels (Table 2).

**Table 2:** Experts’ characteristics.

Phases	Geography	Practical experience	Academic experience	Positions
Phase 1 Propose the HSR Dimensions	Countries: Angola, Australia, Barbados, Brazil, Canada, China, Cyprus, Finland, Germany, India, Ireland, Liberia, Mexico, Mozambique, Netherlands, New Zealand, Nigeria, Portugal, Philippines, Russia, Serbia, Slovenia, Spain, South Africa, United Kingdom, and the United States.	Government: Centers for Disease Control and Prevention, Medicare and Medicaid Services, European Commission, National health departments, or Ministries of Health. NGOs–Commonwealth fund, Public Health Foundation in India, OECD, UK Medical Research Council, UNICEF, WHO (all regional offices), World Bank, and World Economic Forum.	Brazil: Getúlio Vargas Foundation, Oswaldo Cruz Foundation, University of Campinas, and University of São Paulo. International: Grattam Institute, Harvard University, London School of Economics and Political Science, London School of Hygiene and Tropical Medicine, NOVA University of Lisbon, University of Massachusetts Amherst, University of Miami and UTS Business School.	Practical: Advisor, Coordinator, Director, Leader in Economics, Physician, Minister, Representative, and Health secretariat. Academic: Professor, Researcher, and University leadership.
Phase 2 Adapt the HSR dimension and propose/establish indicators	Federal—Brazil. States—Bahia, Minas Gerais, Pernambuco, Rio Grande do Sul, Rio de Janeiro and São Paulo. Municipalities—Aracaju (SE), Belo Horizonte (MG), Campina Grande (PB), Guarulhos (SP), Recife (PE), Rio de Janeiro (RJ) and São Paulo (SP).	Federal: Brazilian Health Regulatory Agency (Anvisa), Health Secretaries and Directories, Ministry of Health, Ministry of Social Insurance, National Agency of Supplementary Health (ANS), National Council of Health Secretaries (Conass), National Council of Municipal Health Secretariats (Conasems), and Senate. State: State government leadership, Health advisor, Health Secretariats, and State Council of Municipal Health Secretariats. Municipality: Health Secretariats. NGO: Institute of Supplementary Health Studies (IESS), National Association of Private Hospitals (ANAHP), National Federation of Health Insurance (FENASAUDE), PAHO (PanAmerican Health Organization), and Union of Hospitals, Clinics, Health Centers and Clinical Analyses and Research Laboratories of the State of São Paulo (SINDHOSP).	Bahia Federal University, Brazilian Association of Collective Health (ABRASCO), Brazilian Center of Health Studies (Cebes), Institute for Applied Economic Research (Ipea), Minas Gerais Federal University, Oswaldo Cruz Foundation, Paraíba Federal University, Pernambuco Federal University, Tiradentes University and University of São Paulo.	Practical: Consultant, Federal Deputy, Federal Health Secretariat, Governor, Minister, Program Director, State and Municipal Health Secretariat, Chairmanship and Hospital Manager. Academic: Professor and Researcher.
Phase 3 Validate the complete HSR framework	Federal: Brazil. State: Rio de Janeiro and São Paulo Municipality: Campina Grande (PB), Carmo de Minas (MG), Rio de Janeiro (RJ), São Bernardo do Campo (SP) and São Vicente (SP)	Federal: Brazilian Health Regulatory Agency (Anvisa), Health secretaries and directories, Ministry of Health, National Council of Health Secretaries (Conass), National Council of Municipal Health Secretariats (Conasems) and Brazilian Company of Hospital Services (EBSERH). State: Health advisor, Health Secretariats, and State Council of Municipal Health Secretariats. Municipality: Health secretariats and hospitals. NGO: PAHO (PanAmerican Health Organization).	Federal University of São Paulo, Oswaldo Cruz Foundation and Paraíba Federal University.	Practical: Executive Secretariat, Federal Health Secretariat, Minister, Program Director, State and Municipal Health Secretariat, Chairmanship, and Hospital Manager. Academic: Professor and Researcher.

Note: all the sample characteristics were listed based on the criteria of at least 2 years of working experience.

**Fig. 2:**
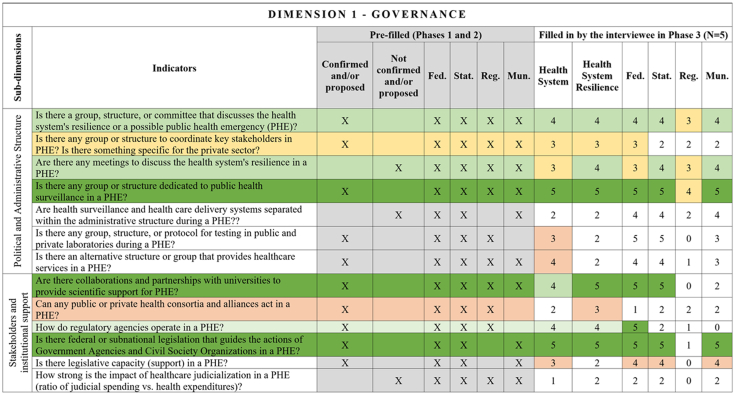
**Governance Dimension–Comprehensive Health System Resilience Framework for Brazil based on the qualitative deductive-inductive approach.** The colors represent the level of agreement in phase 3 experts' answers: dark green—5 answers (full agreement); light green—4 answers (partially full agreement); yellow—3 answers (agreement); and light red—more than 3 answers in the previous phases but did not achieve at least 3 in phase 3.

**Fig. 3:**
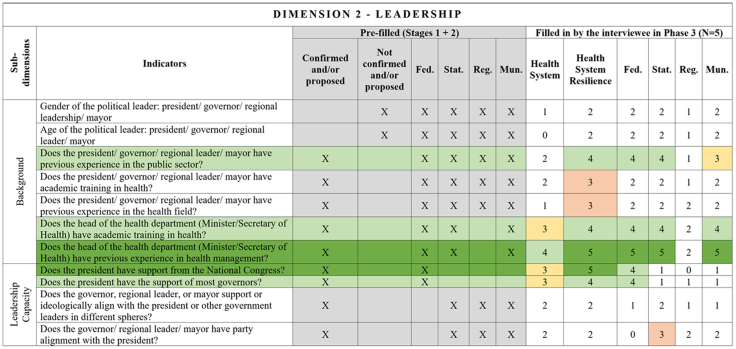
**Leadership dimension–Comprehensive Health System Resilience Framework for Brazil based on the qualitative deductive-inductive approach.** The colors represent the level of agreement in phase 3 experts' answers: dark green—5 answers (full agreement); light green—4 answers (partially full agreement); yellow—3 answers (agreement); and light red—more than 3 answers in the previous phases but did not achieve at least 3 in phase 3.

**Fig. 4:**
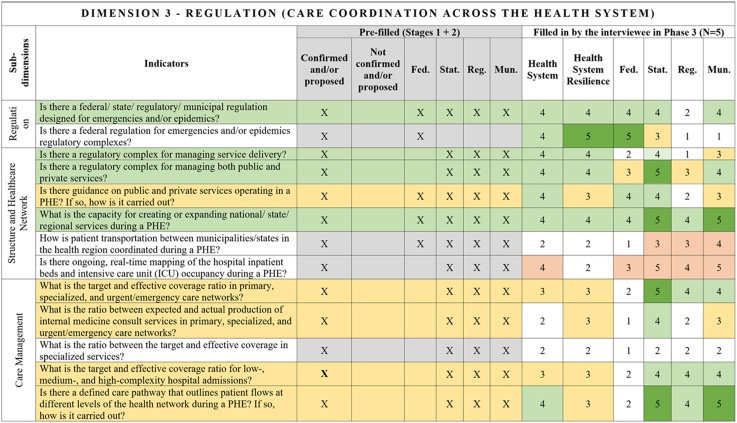
**Regulation, care coordination across the health system, Dimension–Comprehensive Health System Resilience Framework for Brazil based on the qualitative deductive-inductive approach.** The colors represent the level of agreement in phase 3 experts' answers: dark green—5 answers (full agreement); light green—4 answers (partially full agreement); yellow—3 answers (agreement); and light red—more than 3 answers in the previous phases but did not achieve at least 3 in phase 3.

**Fig. 5:**
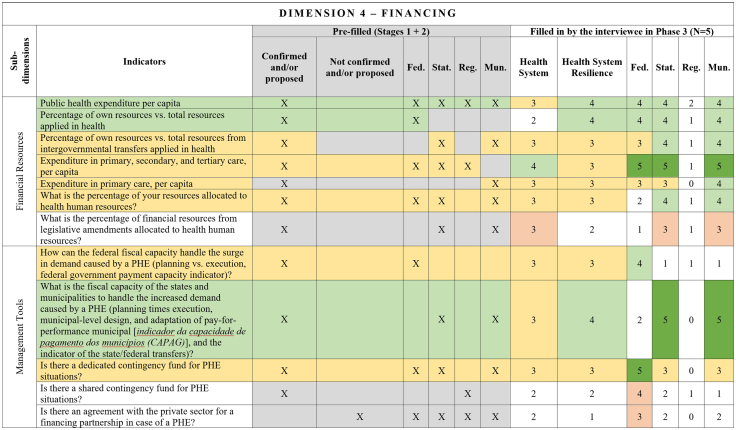
**Financing Dimension–Comprehensive Health System Resilience Framework for Brazil based on the qualitative deductive-inductive approach.** The colors represent the level of agreement in phase 3 experts' answers: dark green—5 answers (full agreement); light green—4 answers (partially full agreement); yellow—3 answers (agreement); and light red—more than 3 answers in the previous phases but did not achieve at least 3 in phase 3.

**Fig. 6:**
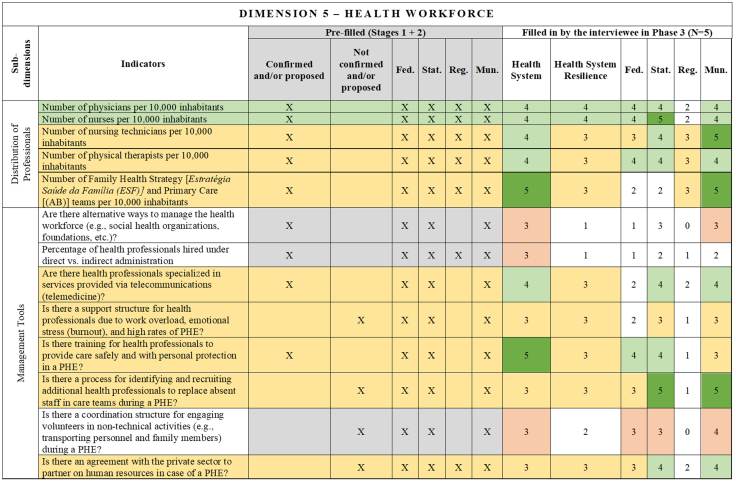
**Health Workforce Dimension–Comprehensive Health System Resilience Framework for Brazil based on the qualitative deductive-inductive approach.** The colors represent the level of agreement in phase 3 experts' answers: dark green—5 answers (full agreement); light green—4 answers (partially full agreement); yellow—3 answers (agreement); and light red—more than 3 answers in the previous phases but did not achieve at least 3 in phase 3.

**Fig. 7:**
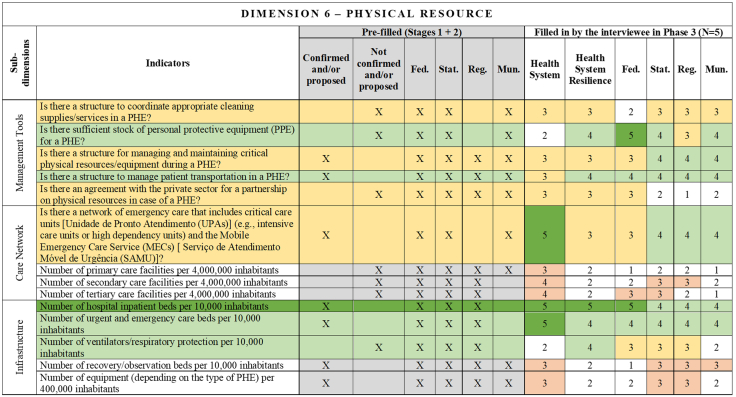
**Physical Resource Dimension–Comprehensive Health System Resilience Framework for Brazil based on the qualitative deductive-inductive approach.** The colors represent the level of agreement in phase 3 experts' answers: dark green—5 answers (full agreement); light green—4 answers (partially full agreement); yellow—3 answers (agreement); and light red—more than 3 answers in the previous phases but did not achieve at least 3 in phase 3.

**Fig. 8:**
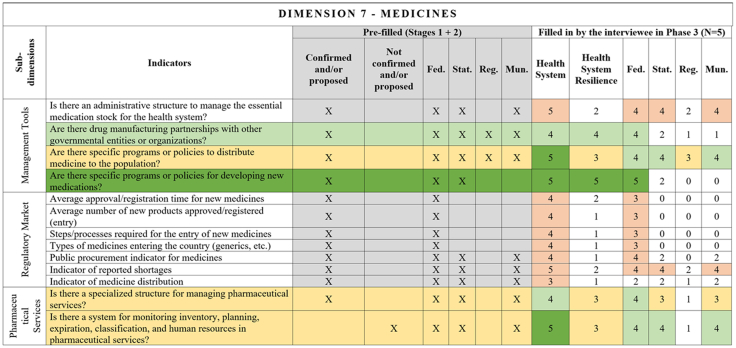
**Medicines Dimension–Comprehensive Health System Resilience Framework for Brazil based on the qualitative deductive-inductive approach.** The colors represent the level of agreement in phase 3 experts' answers: dark green—5 answers (full agreement); light green—4 answers (partially full agreement); yellow—3 answers (agreement); and light red—more than 3 answers in the previous phases but did not achieve at least 3 in phase 3.

**Fig. 9:**
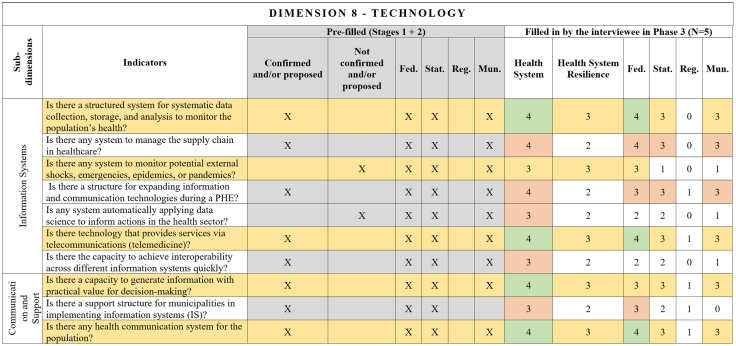
**Technology Dimension–Comprehensive Health System Resilience Framework for Brazil based on the qualitative deductive-inductive approach.** The colors represent the level of agreement in phase 3 experts' answers: dark green—5 answers (full agreement); light green—4 answers (partially full agreement); yellow—3 answers (agreement); and light red—more than 3 answers in the previous phases but did not achieve at least 3 in phase 3.

**Fig. 10:**
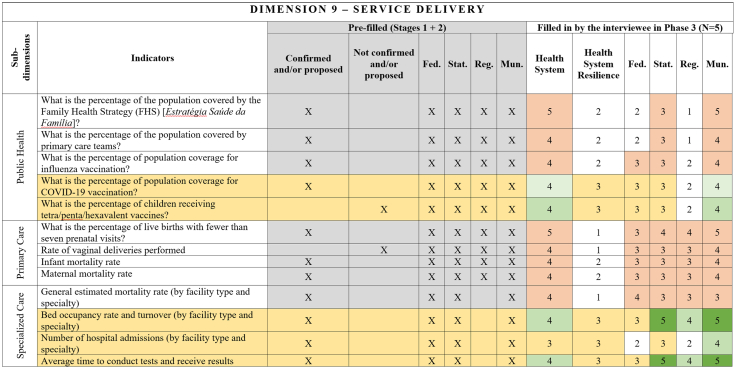
**Service Delivery Dimension–Comprehensive Health System Resilience Framework for Brazil based on the qualitative deductive-inductive approach.** The colors represent the level of agreement in phase 3 experts' answers: dark green—5 answers (full agreement); light green—4 answers (partially full agreement); yellow—3 answers (agreement); and light red—more than 3 answers in the previous phases but did not achieve at least 3 in phase 3.

Fig. 2, Fig. 3, Fig. 4, Fig. 5, Fig. 6, Fig. 7, Fig. 8, Fig. 9, Fig. 10 present the final HSR framework and all the details of the development process. The figures show the results from stages 1 and 2 (grey area) and the sum of the experts' answers from stage 3. To be part of the final HSR framework, the indicator should achieve the criterion of having at least 3 answers in the “Health System Resilience Column”. Also, the same criterion was applied to the “Health System” and “government levels (federal, state, regional and municipal)” columns.

Several key resilience indicators, such as a structure for public health surveillance during PHEs, patient transportation systems, and regulatory frameworks for emergencies and epidemics,^64^ were consistently considered relevant across federal, state, regional, and municipal levels. These indicators reflect system-wide coordination mechanisms that must function cohesively across governance tiers. In the technology dimension, “technology” extends beyond information systems to encompass domestic and regional productive capacity and mechanisms to secure access to critical health technologies.^65^^,^^66^

Other indicators were predominantly assigned to federal-level governance, reflecting central government roles in policy formulation, financing, and strategic coordination.^67^ For example, national emergency regulations, health financing capacity, and agreements with the private sector for physical and human resources were primarily considered federal responsibilities.^68^ Similarly, drug manufacturing partnerships^69^ and national contingency funds were viewed as federal-level responsibilities, as they require centralized planning and resource allocation.

State and municipal governments are crucial for service delivery and local emergency response.^70^ Indicators such as real-time hospital bed occupancy mapping, contingency funds at the state level, and health workforce recruitment strategies during a PHE were more frequently assigned to state and municipal levels. Additionally, indicators measuring local government support for health professionals^71^ and the ability to mobilize emergency care networks^72^ were considered critical at decentralized levels.

Regional-level indicators had the lowest level of agreement among experts, highlighting variability in regional governance structures across different countries and health systems.^73^ For example, while health surveillance and care delivery were considered important at the regional level, indicators related to political and administrative leadership and regulatory agency coordination were less agreed upon. This reflects structural differences in regional governance capacity, which can vary significantly between federalized and centralized health systems.^74^

The differentiation of indicators across government levels advances the discussion on multi-level governance in HSR frameworks, emphasizing the need for vertical integration and coordination among national, subnational, regional and local actors.^75^ While federal-level planning and regulation provide strategic direction,^76^ state^67^ and municipal governments are crucial for operational resilience, particularly in service delivery, emergency response, and localized adaptation.^77^

## Discussion

Compared to previous HSR frameworks, this study introduces new elements that advance the field by incorporating multi-level governance, adaptability indicators, and system-wide coordination mechanisms. We position resilience atop structural preconditions–coverage, public financing, and strong primary health care–while focusing measurement on capacities that modulate performance during shocks.^24^^,^^53^ This avoids conflating steady-state performance with dynamic response.^78^ Also, we specify actionable levers–equity-oriented resource allocation rules, fiscal safeguards (e.g., contingency funds), intergovernmental compacts, surge workforce mechanisms, and technology-access strategies–to translate measurement into policy.

COVID-19 reaffirmed that social contracts sustaining health investment, integrity of public institutions, and the production and use of scientific evidence are enabling conditions for resilience.^79^ We incorporate governance coherence, evidence-informed decision-making, and transparent communication as cross-cutting elements, operationalised via indicators on surveillance, emergency regulation, and rapid learning.^80^

Referred with PAHO's fuzzy-logic approach to potential resilience^81^ and indices such as the GHS Index,^42^ our contribution is: (*i*) a Brazil-specific, interview-validated indicator set; (*ii*) explicit allocation across federal, state, regional, and municipal levels; and (*iii*) a delineation of resilience capacities versus general performance metrics. While fuzzy-logic scoring provides probabilistic aggregation, our framework prioritises governance assignment and implementation guidance. Finally, whereas Building Blocks and related roadmaps emphasise operational readiness,^3^^,^^8^^,^^82^^,^^83^ our contribution is a policy-to-operations bridge, which assigns responsibility by governance level, includes fiscal safeguards and coordination mechanisms, and differentiates steady-state performance from adaptive capacity in decentralised contexts.

A major contribution of this framework is incorporating indicators contextualized across different levels of governance–federal, state, regional, and municipal.^84^ The results indicate that some indicators were broadly applicable across all levels, while others were specific to certain levels of government.^85^

One of the most notable advancements in this framework is the explicit distinction between health system and health system resilience indicators, which was not always clear in previous studies.^86^^,^^87^ While prior frameworks, such as the WHO's “building blocks” model, emphasized health system inputs (e.g., financing, infrastructure, and human resources), they did not sufficiently address adaptive capacities needed for crisis response.^84^

Additionally, this study refines resilience measurement by considering the role of governance structures at multiple levels, whereas previous resilience frameworks primarily focused on national-level resilience.^6^ Including state, regional, and municipal perspectives ensures a more granular and actionable understanding of resilience in decentralized health systems^88^ like Brazil's Unified Health System (SUS).^86^^,^^87^

Another key innovation is the health workforce adaptability indicators, such as mechanisms for recruiting additional personnel, burnout support, and telemedicine integration. These aspects were not systematically included in earlier HSR frameworks but have proven to be critical during public health emergencies like COVID-19.^36^^,^^89^ The ability to scale up health workforce capacity in real-time is a crucial resilience function that distinguishes this framework from previous models primarily focused on static workforce indicators (e.g., physician-to-population ratios).^90^

Finally, this study advances the literature by integrating real-time system monitoring indicators, including hospital bed occupancy mapping and data-driven decision-making tools. Previous HSR frameworks lacked dynamic, real-time indicators, often relying on retrospective health system performance data.^4^^,^^78^ By incorporating technological and surveillance indicators, this framework strengthens proactive resilience planning and crisis responsiveness. Resilience during COVID-19 was constrained by technological and productive asymmetries. We therefore include indicators on access and surge production to reflect health economic-industrial capacity relevant to decentralised systems.^66^

Beyond assessment, the indicator set can inform analyses of how political economy, fiscal federalism, and institutional legacies shape resilience. Future work will link indicator profiles to temporal changes in governance and financing to understand structural determinants of resilience. These advancements contribute to a more comprehensive and actionable resilience framework that can better inform policymakers, particularly in multi-institutional and decentralized health systems.

## Health system versus health system resilience framework

One of the key aspects of developing a Health System Resilience (HSR) framework is distinguishing between indicators that measure the general performance of a health system and those that assess its resilience to shocks and public health emergencies (PHEs).^86^^,^^87^ In our analysis, several indicators commonly used in health system evaluations were not included in the final HSR framework.

The health system financing indicators focusing on routine expenditures and resource distribution did not fully translate into resilience metrics.^78^ For instance, public health expenditure per capita and the percentage of own resources applied in health were included in the health system dimension^91^ but were not prioritized for HSR, as they do not necessarily reflect adaptability in crisis situations.^41^ Similarly, indicators related to health system coverage, such as the PHC indicators, are important for service delivery^80^ but do not inherently measure resilience, as they do not capture system flexibility during a PHE. Another group of indicators that did not enter the HSR framework relates to service delivery outcomes, including infant mortality rate, maternal mortality rate, and general estimated mortality rate by facility type and speciality. While these indicators provide a strong assessment of a health system's performance,^92^ they are lagging indicators and do not necessarily inform how well the system can respond to sudden shocks.

Several indicators traditionally used to assess market stability and efficiency in the medicines and regulatory market domain, such as approval time for new medicines, the number of new pharmaceutical products registered, and public procurement efficiency,^93^^,^^94^ were not incorporated into the final HSR framework. Although these factors influence long-term health system effectiveness, they do not immediately predict system resilience in responding to crises.

The exclusion of these indicators reflects a significant methodological distinction: while a strong health system provides the foundation for resilience,^24^ the HSR framework prioritizes adaptive, absorptive, and transformative capacities^7^^,^^95^–factors determining a system's ability to withstand and recover from crises.

## How to use the framework in practice

This framework is designed for decision-makers at federal, state, regional, and municipal levels to diagnose resilience gaps, prioritize actions, and monitor progress. It operationalizes the nine dimensions, 18 subdimensions, and 65 indicators validated in Fig. 2, Fig. 3, Fig. 4, Fig. 5, Fig. 6, Fig. 7, Fig. 8, Fig. 9, Fig. 10, with governance responsibilities allocated.

### Step 1–Scope (define the use case and level)

Specify the governance level(s) and dimensions to be assessed. Select the most relevant subdimensions/indicators for that scope, using Fig. 2, Fig. 3, Fig. 4, Fig. 5, Fig. 6, Fig. 7, Fig. 8, Fig. 9, Fig. 10 to identify where responsibility primarily lies and where coordination is required across levels.

### Step 2–Map (establish the baseline)

Compile brief evidence for each selected indicator: current policies/regulations, financing rules, workforce arrangements, assets, and recent performance during stressors. Sources include routine administrative data, surveillance feeds, policy documents, and after-action reports. Note equity-relevant heterogeneity (e.g., variation across regions/municipalities).

### Step 3–Score (rate presence/strength 0–3 with evidence)

Use a simple, anchored scale applied consistently across levels:

0 Absent: No policy/instrument/mechanism; ad hoc responses only.
1 Emerging: Policy/instrument exists on paper or in pilot; partial implementation; limited coverage or capacity; not stress-tested.
2 Established: Implemented with defined processes and coverage; demonstrated function in at least one stressor; known gaps remain.
3 Institutionalized: Routinely implemented at scale; financed and governed with clear accountability; stress-tested and improved via learning cycles.

Each score should cite the specific evidence used (policy name/date, dataset, recent activation). For reliability, dual-rate a 10–20% subsample and reconcile discrepancies.

### Step 4–Prioritize (choose high-leverage actions)

Create a short priority matrix for scored indicators using four lenses: current score (focus on ≤1), leverage (breadth of effect/system-wide coordination potential), equity impact (expected reduction in territorial or socioeconomic disparities), and feasibility within 6–12 months. Where helpful, flag a minimal “critical set” of must-have capacities that should not fall below 2. Outputs are 3–6 priority items with brief justifications.

### Step 5–Plan (match gaps to policy levers and assign accountability)

For each priority item, specify the main lever(s) and the accountable level(s), aligned to the dimensions in Fig. 2, Fig. 3, Fig. 4, Fig. 5, Fig. 6, Fig. 7, Fig. 8, Fig. 9, Fig. 10. Define deliverables, timelines (quarterly), and milestones, plus required cross-level coordination.

### Step 6–Monitor and learn (quarterly review with triggers)

Set quarterly re-scoring and track a compact dashboard of leading indicators. Use red–amber–green (RAG) thresholds, document corrective actions, and update responsibilities if gaps persist. After real stressors, conduct short after-action reviews and integrate lessons into the next cycle.

## Conclusions

This study presents a novel Health System Resilience (HSR) framework and provides a structured and context-sensitive approach to assessing resilience in decentralized health systems by distinguishing between general and resilience-specific health system indicators. It highlights the importance of governance structures across institutional levels, ensuring that resilience planning considers different levels of government and their capacities and responsibilities. Although tailored to SUS, the development logic and indicator families can generalise to other decentralised systems. Adaptation requires: (*i*) mapping governance responsibilities; (*ii*) re-allocating indicators to local tiers; and (*iii*) calibrating policy levers to national financing rules.

Therefore, the study significantly contributes to the scientific community, society, and policymakers. Academically, it refines resilience measurement methodologies by integrating dynamic indicators, such as real-time system monitoring, health workforce adaptability, and emergency regulatory frameworks. These insights enhance resilience assessment tools, making them more applicable to low- and middle-income countries (LMICs) with decentralized health governance. For society, a well-defined HSR framework strengthens preparedness and response strategies, ultimately improving health system responsiveness to PHEs. For policymakers, this study provides an evidence-based structure to inform resilience-building initiatives, ensuring that practical and measurable indicators guide decision-making.

## Limitations

This study has some limitations. First, even with a unique group of HSR experts participating, the convenience of the interviewee selection may have influenced the results of the HSR framework in terms of the inclusion and exclusion of specific indicators. Second, the variation in the interviews period of time, 22 months, across the study phases could have led to different perspectives as resilience and COVID-19 discussions evolved over time. Third, the framework may risk emphasising measurable indicators over deeper structural reforms (e.g., financing architecture, industrial policy). We mitigate this by locating indicators within a broader reform agenda and urging application alongside structural diagnostics. Also, we did not estimate inter-rater reliability or derive indicator weights. Future work should pilot the scoring template in multiple states/regions, estimate reliability (e.g., Gwet's AC1), explore weighting via Delphi or multicriteria methods, and model associations with service continuity and outcomes.

## Contributors

All authors conceived the study; the data curation and formal analysis were executed by MACP; MCC was the main responsible for funding and resources acquisition, followed by the other authors; MP led the investigation, methodology and project administration parts, followed by the other authors; the software was managed by MACP; MCC, RR and AM supervised all the steps, and all the authors validated, visualized and wrote (original, review and editing) the draft and final versions.

## Declaration of interests

MACP, EAL, RR, AM and MCC declare no competing interests.

## Appendix A Supplementary data

Appendix 4

Resumen ESP

Resumo POR
