# Supplementary material for: Building a health system resilience framework: national, state, regional, and local perspectives
Source: Lancet Reg Health Am. 2025 Dec 11;54:101334. doi: 10.1016/j.lana.2025.101334 (PMC12757546; doi:10.1016/j.lana.2025.101334)
Supplement: Appendix 1 [file mmc1.docx]

INTERVIEW SCRIPT – STAGE 1

Style: semi-structured interview, with data to be collected from this interview script.

Target group: national and international experts in the area of “resilience of healthcare systems”.

Objective: define the dimensions that should be incorporated into the analysis framework of a healthcare system.

1. What defines resilience of a healthcare system?

2. If you were to propose a comprehensive framework to analyze the resilience of a healthcare system, which dimensions would it include?

3. Based on the resilience model of a health system created by the World Health organization in 2010 (WHO, 2010), revisited in a recent scientific publication in the journal “Nature Medicine” (Haldane et al., 2021), would you add some of these dimensions?***

a) Governance and Leadership: ( ) b) Resources (financial, human and physical): ( ) c) Medicine and Technology: ( ) d) Services provision: ( )

**interviewer: in case the interviewee has already elaborated on these dimensions, jump to question 4. In cases where only some of these dimensions have been elaborated on, ask the question citing only the missing dimensions.*

4. How would you measure the dimensions of such a comprehensive framework? Are there specific indicators that should be included?

*References*

Haldane, V., De Foo, C., Abdalla, S.M. et al. (2021). Health systems resilience in managing the COVID-19 pandemic: lessons from 28 countries. *Nat Med,* 27, 964–980. DOI: [https://doi.org/10.1038/s41591-021-01381-y](about:blank)

WHO. World Health Organization. (2010). Monitoring the building blocks of health systems: a handbook of indicators and their measurement strategies. Available at: [https://www.who.int/workforcealliance/knowledge/toolkit/26.pdf](about:blank)
