# Supplementary material for: Building a health system resilience framework: national, state, regional, and local perspectives
Source: Lancet Reg Health Am. 2025 Dec 11;54:101334. doi: 10.1016/j.lana.2025.101334 (PMC12757546; doi:10.1016/j.lana.2025.101334)
Supplement: Appendix 2 [file mmc2.docx]

INTERVIEW SCRIPT – STAGE 2

Style: semi-structured interview, with data to be collected based on this interview script.

Target Group: national experts* in one of the following “Health System Resilience” areas: Governance, Regulation, Leadership, Financing; Health Workforce, Physical Resources, Medicines, Technology, Service Delivery, and Supplementary Insurance**

Objective: to define the indicators that make up the dimensions (defined in Stage 1) of the framework for analyzing the resilience of a health system at the federal, state, regional, and municipal levels

**Each expert will answer the questions referring to the dimension they specialize in, i.e., only one of the sections listed below.*

***If any dimension not mentioned above is identified in Stage 1, a national expert will be included.*

Section 1 – Governance

1. How does the Governance dimension fit within the resilience of the Brazilian health system? How might it be defined, and which aspects should it comprise?

2. What indicators can measure the Governance dimension? Are these indicators already calculated? If so, using variables from which databases? If not, how might they be created?

3. Based on the literature review conducted by the research, would you like to add any of these indicators to the Governance dimension? ***

a) is there any group, structure, or committee that discusses the health system's resilience or a possible public health emergency (PHE)? ( ) b) is there federal or subnational legislation that guides the actions of Government Agencies and Civil Society Organizations in a PHE? ( ) c) is there any group or structure to coordinate key stakeholders in a PHE? ( ) d) is there any meeting to discuss the health system's resilience in a PHE? ( ) e) is there any group or structure dedicated to public health surveillance in a PHE? ( ) f) are public health surveillance and health care delivery systems separated within the administrative structure during a PHE? ( ) g) is there any group, structure, or protocol for testing in public and private laboratories during a PHE? ( ) h) are there collaborations and partnerships with universities to provide scientific support for PHE? ( ) i) can any public or private health consortia and alliances act in a PHE? ( ) j) is there a dedicated fund for the health sector to address a PHE? ( ) l) is there an alternative structure or group that provides healthcare services in a PHE? ( ) m) is there a structure to coordinate the private health sector in a PHE? ( ) n) how do regulatory agencies operate in a PHE? o) how does the healthcare judicialization (i.e., legal/judicial interventions) impact the system’s response to a PHE?

*** Interviewer’s note: if the interviewee has already listed these indicators, skip to question 4. If only some indicators were mentioned, ask only about the missing ones.*

4. As an expert, would you like to add any additional information to create the Governance dimension? Is there any indicator that has not been mentioned yet?

Section 2 – Leadership

1. How does the Leadership dimension fit within the resilience of the Brazilian health system? How might it be defined, and which aspects should comprise it?

2. What indicators can measure the Leadership dimension? Do these indicators already exist? If so, using variables from which databases? If not, how might they be created?

3. Based on the literature review conducted by the research, would you like to add any of these indicators to the Leadership dimension?***

a) what is the gender of the political leader? ( ) b) how old is the political leader? ( ) c) what is the educational background of the political leader? ( ) d) does the political leader have previous experience in the public sector? ( ) e) does the political leader have academic training in health? ( ) f) does the political leader have previous experience in the health field? ( ) g) does the head of the health department have academic training in health? ( ) h) does the head of the health department have previous experience in health management? ( ) i) does the chief executive have ideological alignment with the legislative branch? ( ) j) does the chief executive have support from the legislative branch? ( ) k) does the chief executive have support or ideological alignment with other government leaders in different spheres? ( ) l) does the chief executive have support from other government leaders in different spheres? ( )

*** Interviewer’s note: if the interviewee has already listed these indicators, skip to question 4. If only some indicators were mentioned, ask only about the missing ones.*

4. As an expert, would you like to add any additional information to create the Leadership dimension? Is there any indicator that has not been mentioned yet?

Section 3 – Regulation (Care coordination across the health system)

1. How does the Regulation (Care coordination across the health system)’s dimension fit within the resilience of the Brazilian health system? Considering the regulatory complex and care coordination across the healthcare system, how might it be defined?

2. What indicators can measure the Regulation/Care coordination across the healthcare system's dimensions? Do these indicators already exist? If so, using variables from which databases? If not, how might they be created?

3. Based on the literature review conducted by the research, would you like to add any of these indicators to the Regulations/Care coordination across the healthcare system’s dimension?***

a) is there regulation geared towards responding to emergencies and/or epidemics? ( ) b) is there a regulatory complex for managing service delivery? ( ) c) what is the target and effective coverage ratio in primary, specialized, and urgent/emergency care networks? ( ) d) what is the ratio between expected and actual production of internal medicine consult services in primary, specialized, and urgent/emergency care networks? ( ) e) what is the ratio between the target and effective coverage in specialized services? ( ) f) what is the target and effective coverage ratio for low-, medium-, and high-complexity hospital admissions? ( ) g) is there a defined care pathway that outlines patient flows at different levels of the health network during a PHE? if so, how is it carried out? h) is there guidance on public and private services operating in a PHE? if so, how is it carried out? i) what is the capacity for creating or expanding national and regional services during a PHE? j) how is patient transportation between municipalities/states in the health region coordinated during a PHE? k) is there ongoing, real-time mapping of the hospital inpatient beds and intensive care unit (ICU) occupancy during a PHE? if so, how is it conducted?

*** Interviewer’s note: if the interviewee has already listed these indicators, skip to question 4. If only some indicators were mentioned, ask only about the missing ones.*

4. As an expert, would you like to add any additional information to create the Regulations dimension? Is there any indicator that has not been mentioned yet?

Section 4 - Financing

1. How does the Financing dimension fit within the resilience of the Brazilian health system? How might it be defined, and which aspects should comprise it?

2. What indicators can measure the Financing dimension? Do these indicators already exist? If so, using variables from which databases? If not, how might they be created?

3. Based on the literature review conducted by the research, would you like to add any of these indicators to the Grants and Funding dimension?***

a) public health expenditure per capita ( ) b) ratio of “percentage of own resources vs. resources from intergovernmental transfers” applied in health ( ) c) expenditures in primary, secondary, and tertiary care, per capita ( ) d) percentage of own resources allocated to health human resources ( )
e) is there a dedicated contingency fund for emergency situations, such as a pandemic? ( ) f) is there financial support from the private sector through public-private partnerships? ( ) g) what is the fiscal capacity of the states and municipalities to handle the increased demand caused by a PHE (planning times execution, municipal-level design, and adaptation of pay-for-performance municipal [*indicador da capacidade de pagamento dos municípios (CAPAG)*], and the indicator of the state/federal transfers)? ( )

*** Interviewer’s note: if the interviewee has already listed these indicators, skip to question 4. If only some indicators were mentioned, ask only about the missing ones.*

4. As an expert, would you like to add any additional information to create the Finance dimension? Is there any indicator that has not been mentioned yet?

Section 5 – Health Workforce

1. How does the Health Workforce dimension fit within the resilience of the Brazilian health system? How might it be defined, and which aspects should comprise it?

2. What indicators can measure the Health Workforce dimension? Do these indicators already exist? If so, using variables from which databases? If not, how might they be created?

3. Based on the literature review conducted by the research, would you like to add any of these indicators to the Human Resources dimension?***

a) number of physicians per 10,000 inhabitants ( ) b) number of nurses per 10,000 inhabitants ( )
c) number of nursing technicians per 10,000 inhabitants ( ) d) number of physical therapists per 10,000 inhabitants ( ) e) number of Family Health Strategy [Estratégia Saúde da Família (ESF)] and Primary Care [(AB)] teams per 10,000 inhabitants ( ) f) are there alternative ways to manage the health workforce (e.g., social health organizations, foundations, etc.)? Which ones? ( ) g) percentage of health professionals hired under direct vs. indirect administration ( ) h) are there health professionals specialized in services provided via telecommunications (telemedicine)? ( ) i) is there a support structure for health professionals due to work overload, emotional stress (burnout), and high rates of PHE? If so, how is it provided? ( ) j) is there training for health professionals to provide care safely and with personal protection in a PHE? If so, how is it carried out? ( ) k) is there a process to identify and recruit additional health professionals to replace absent staff in care teams during a PHE? ( ) l) is there a coordination structure for engaging volunteers in non-technical activities (e.g., transporting personnel and family members) during a PHE? ( )

*** Interviewer’s note: if the interviewee has already listed these indicators, skip to question 4. If only some indicators were mentioned, ask only about the missing ones.*

4. As an expert, would you like to add any additional information to create the Health Workforce dimension? Is there any indicator that has not been mentioned yet?

Section 6 – Physical Resources

1. How does the Physical Resources dimension fit within the resilience of the Brazilian health system? How might it be defined, and which aspects should comprise it?

2. What indicators can measure the Physical Resources dimension? Do these indicators already exist? If so, using variables from which databases? If not, how might they be created?

3. Based on the literature review conducted by the research, would you like to add any of these indicators to the Physical Resources dimension?***

a) number of hospital inpatient beds per 10,000 inhabitants ( ) b) number of urgent and emergency care beds per 10,000 inhabitants ( ) c) number of ventilators/respiratory protection per 10,000 inhabitants ( ) d) number of recovery/observation beds per 10,000 inhabitants ( ) e) is there a network of emergency care that includes critical care units [Unidade de Pronto Atendimento (UPAs)] (e.g. intensive care units or high dependency units) and the Mobile Emergency Care Service (MECs) [ Serviço de Atendimento Móvel de Urgência (SAMU)]? ( ) f) number of primary care facilities per 1,000,000 inhabitants ( ) g) number of secondary care facilities per 1,000,000 inhabitants ( ) h) number of tertiary care facilities per 1,000,000 inhabitants ( ) i) number of specific equipment (depending on type) per 100,000 inhabitants ( ) j) is there a structure to coordinate appropriate cleaning supplies/services in a PHE? ( ) k) is there a sufficient stock of personal protective equipment (PPE) for a PHE? If so, how is this measured? ( ) l) is there a structure for managing and maintaining critical physical resources/equipment during a PHE? ( )

*** Interviewer’s note: if the interviewee has already listed these indicators, skip to question 4. If only some indicators were mentioned, ask only about the missing ones.*

4. As an expert, would you like to add any additional information to create the Physical Resources dimension? Is there any indicator that has not been mentioned yet?

Section 7 – Medicines

1. How does the Medicines dimension fit within the resilience of the Brazilian health system? How might it be defined, and which aspects should comprise it?

2. What indicators can measure the Medicines dimension? Do these indicators already exist? If so, using variables from which databases? If not, how might they be created?

3. Based on the literature review conducted by the research, would you like to add any of these indicators to the Medicines dimension?***

a) is there an administrative structure to manage the essential stock of medication for the health system? ( ) b) are there drug manufacturing partnerships with other governmental entities or organizations? ( ) c) are there specific programs or policies to distribute medicine to the population? ( ) d) are there specific programs or policies for developing new medications? ( )

*** Interviewer’s note: if the interviewee has already listed these indicators, skip to question 4. If only some indicators were mentioned, ask only about the missing ones.*

4. As an expert, would you like to add any additional information to create the Medicine dimension? Is there any indicator that has not been mentioned yet?

Section 8 – Technology

1. How does the Technology dimension fit within the resilience of the Brazilian health system? How might it be defined, and which aspects should comprise it?

2. What indicators can measure the Technology dimension? Do these indicators already exist? If so, using variables from which databases? If not, how might they be created?

3. Based on the literature review conducted by the research, would you like to add any of these indicators to the Technology dimension?***

a) is there any system to monitor the population’s health? ( ) b) is any system automatically applying data science to inform actions in the health sector? ( ) c) is there technology that provides services via telecommunications (telemedicine)? ( ) d) is there any system to manage the supply chain in healthcare? ( ) e) is there any system to monitor potential external shocks, emergencies, epidemics, or pandemics? ( ) f) is there a structure for expanding information and communication technologies in the event of a PHE? ( )

*** Interviewer’s note: if the interviewee has already listed these indicators, skip to question 4. If only some indicators were mentioned, ask only about the missing ones.*

4. As an expert, would you like to add any additional information to create the Technology dimension? Is there any indicator that has not been mentioned yet?

Section 9 – Service Delivery

1. How does the Service Delivery dimension fit within the resilience of the Brazilian health system? How might it be defined, and which aspects should comprise it?

2. What indicators can measure the Service Delivery dimension? Do these indicators already exist? If so, using variables from which databases? If not, how might they be created?

3. Based on the literature review conducted by the research, would you like to add any of these indicators to the Health Service Delivery dimension?***

a) what is the percentage of the population that had medical consultations in the last 12 months? ( ) b) what is the percentage of the population covered by the Family Health Strategy (FHS) [*Estratégia Saúde da Família*]? ( ) c) what is the percentage of the population covered by primary care teams? ( ) d) percentage of live births with fewer than seven prenatal visits? ( ) e) what is the rate of hemodialysis procedures performed? ( ) f) what is the percentage of hospitalizations for ambulatory care-sensitive conditions (ACSCs)? ( ) g) what is the rate of medium- and high-complexity procedures performed? ( ) h) what is the percentage of population coverage for influenza vaccination? ( ) i) what is the rate of medium- and high-complexity clinical and surgical procedures performed? ( ) j) what is the percentage of population coverage for COVID-19 vaccination? ( ) k) what is the rate of medium- and high-complexity hospital admissions performed? ( ) l) what is the percentage of children receiving tetra/penta/hexavalent vaccines? ( ) m) what is the rate of vaginal deliveries performed? ( )

*** Interviewer’s note: if the interviewee has already listed these indicators, skip to question 4. If only some indicators were mentioned, ask only about the missing ones.*

4. As an expert, would you like to add any additional information to create the Service Delivery dimension? Is there any indicator that has not been mentioned yet?

Section 10 – Supplementary Insurance

1. How does Supplementary Insurance fit within the resilience of the Brazilian health system? How might it be defined, and which aspects should it comprise?

2. How does Supplementary Insurance align with the dimensions of “Governance, Regulation, Leadership, Financing, Health Workforce, Physical Resources, Medicines, Technology, and Service Delivery”?

3. Which indicators can measure Supplementary Insurance? Are they different from those used in the Governance, Regulation, Leadership, Financing, Health Workforce, Physical Resources, Medicines, Technology, and Service Delivery dimensions? If so, using variables from which databases? If not, how might they be created?
