# Supplementary material for: Building a health system resilience framework: national, state, regional, and local perspectives
Source: Lancet Reg Health Am. 2025 Dec 11;54:101334. doi: 10.1016/j.lana.2025.101334 (PMC12757546; doi:10.1016/j.lana.2025.101334)
Supplement: Appendix 3 [file mmc3.docx]

INTERVIEW SCRIPT – STAGE 3

Style: semi-structured interview, with data to be collected based on this interview script.

Target Group: national experts with extensive experience in health systems through their role as managers within the Brazilian Unified Health System [*Sistema Único de Saúde* (SUS)].

Objective: to validate/test/propose the indicators that make up the dimensions (defined in Stages 1 and 2) of the framework for analyzing the resilience of the Brazilian health system at the federal, state, regional, and municipal levels.

Interview script guide

Dear interviewee, clearly and objectively, you will place an “X” in the five columns on the right of each dimension table under the “Filled in by the interviewee” heading. However, to do so, you need to understand the rationale behind this work. *The framework for analyzing the resilience of the Brazilian health system* is divided into the following dimensions: *Governance, Leadership, Regulation (care coordination across the health system), Financing, Human Resources, Physical Resources, Medicines, Technology, and Service Delivery*. Indicators (column 2) are organized into sub-dimensions (column 1) within each dimension.

These indicators, sub-dimensions, and dimensions were defined in two previous stages based on the literature and semi-structured interviews with health system and resilience experts worldwide and in Brazil (federal, state, and municipal levels). Hence, in the “Pre-filled (Stages 1 + 2)” section, you will see check marks (“X”) that have already been placed. You will *not* modify those. The meaning of each column is as follows:

- *Confirmed and/or proposed (column 3)* – indicates that the indicator was confirmed and/or proposed in Stages 1 and 2;
- *Not Confirmed and/or proposed (column 4)* – indicates that the indicator was not confirmed and/or proposed in Stages 1 and 2 but did appear in the literature;
- *Fed., State, Reg., Mun. (columns 5 to 8)* – referring to the Federal, State, Regional, and Municipal levels, marking that the indicators should be measured and analyzed at that specific governmental or (in the case of Regional) normative level.

Based on that background and your expertise, you *will* place an “X” in the five columns to the right in the “Filled in by the interviewee” section. The meanings of these columns are:

- *Health System (column 9)* – indicates that the indicator should be part of the overall framework for analyzing the Brazilian health system (but not necessarily its resilience);
- *Health System Resilience (column 10)* – indicates that the indicator should *necessarily* be part of the framework for analyzing the resilience of the Brazilian health system;
- *Fed., State, Reg., Mun. (columns 11 to 14)* – referring to the Federal, State, Regional, and Municipal levels, indicating that the indicators should be measured and analyzed at that governmental (or regional) level.

Within each dimension’s table, and again at the end of the script, you will find spaces for “Comments.” Feel free to criticize the dimensions, explain your choices, and/or propose new indicators. When you finish the script, please save the document and email it back to us.

*Note:* We use the following definition of health system resilience: *“the ability to prepare for, manage (absorb, adapt, and transform), and learn from shocks, with a context-based understanding.”*

Dimension 1 - Governance

| Sub-dimensions | Indicators | Pre-filled (Stages 1 + 2) | | | | | | Filled in by the interviewee | | | | | |  |
| --- | --- | --- | --- | --- | --- | --- | --- | --- | --- | --- | --- | --- | --- | --- |
|  |  | Confirmed and/or proposed | Not confirmed and/or proposed | Fed. | Stat. | Reg. | Mun. | Health System | Health System Resilience | Fed. | Stat. | Reg. | Mun. |  |
| Political and Administrative Structure | Is there a group, structure, or committee that discusses the health system's resilience or a possible public health emergency (PHE)? | X |  | X | X | X | X | 4 | 4 | 4 | 4 | 3 | 4 |  |
|  | Is there any group or structure to coordinate key stakeholders in PHE? Is there something specific for the private sector? | X |  | X | X | X | X | 3 | 3 | 3 | 2 | 2 | 2 |  |
|  | Are there any meetings to discuss the health system's resilience in a PHE? |  | X | X | X | X | X | 3 | 4 | 3 | 4 | 3 | 4 |  |
|  | Is there any group or structure dedicated to public health surveillance in a PHE? | X |  | X | X | X | X | 5 | 5 | 5 | 5 | 4 | 5 |  |
|  | Are health surveillance and health care delivery systems separated within the administrative structure during a PHE?? |  | X | X | X |  | X | 2 | 2 | 4 | 4 | 2 | 4 |  |
|  | Is there any group, structure, or protocol for testing in public and private laboratories during a PHE? | X |  | X | X | X |  | 3 | 2 | 5 | 5 | 0 | 3 |  |
|  | Is there an alternative structure or group that provides healthcare services in a PHE? | X |  | X | X | X | X | 4 | 2 | 4 | 4 | 1 | 3 |  |
| Stakeholders and institutional support | Are there collaborations and partnerships with universities to provide scientific support for PHE? | X |  | X | X | X | X | 4 | 5 | 5 | 5 | 0 | 2 |  |
|  | Can any public or private health consortia and alliances act in a PHE? | X |  | X | X | X |  | 2 | 3 | 1 | 2 | 2 | 2 |  |
|  | How do regulatory agencies operate in a PHE? | X |  | X | X | X |  | 4 | 4 | 5 | 2 | 1 | 0 |  |
|  | Is there federal or subnational legislation that guides the actions of Government Agencies and Civil Society Organizations in a PHE? | X |  | X | X |  | X | 5 | 5 | 5 | 5 | 1 | 5 |  |
|  | Is there legislative capacity (support) in a PHE? | X |  | X | X |  | X | 3 | 2 | 4 | 4 | 0 | 4 |  |
|  | How strong is the impact of healthcare judicialization in a PHE (ratio of judicial spending vs. health expenditures)? |  | X | X | X | X | X | 1 | 2 | 2 | 2 | 0 | 2 |  |
| Comments: | | | | | | | | | | | | | | |

Dimension 2 - Leadership

| Sub-dimensions | Indicators | Pre-filled (Stages 1 + 2) | | | | | | Filled in by the interviewee | | | | | |  |
| --- | --- | --- | --- | --- | --- | --- | --- | --- | --- | --- | --- | --- | --- | --- |
|  |  | Confirmed and/or proposed | Not confirmed and/or proposed | Fed. | Stat. | Reg. | Mun. | Health System | Health System Resilience | Fed. | Stat. | Reg. | Mun. |  |
| Background | Gender of the political leader: president/ governor/ regional leadership/ mayor |  | X | X | X | X | X | 1 | 2 | 2 | 2 | 1 | 2 |  |
|  | Age of the political leader: president/ governor/ regional leader/ mayor |  | X | X | X | X | X | 0 | 2 | 2 | 2 | 1 | 2 |  |
|  | Does the president/ governor/ regional leader/ mayor have previous experience in the public sector? | X |  | X | X | X | X | 2 | 4 | 4 | 4 | 1 | 3 |  |
|  | Does the president/ governor/ regional leader/ mayor have academic training in health? | X |  | X | X | X | X | 2 | 3 | 2 | 2 | 1 | 2 |  |
|  | Does the president/ governor/ regional leader/ mayor have previous experience in the health field? | X |  | X | X | X | X | 1 | 3 | 2 | 2 | 2 | 2 |  |
|  | Does the head of the health department (Minister/Secretary of Health) have academic training in health? | X |  | X | X |  | X | 3 | 4 | 4 | 4 | 2 | 4 |  |
|  | Does the head of the health department (Minister/Secretary of Health) have previous experience in health management? | X |  | X | X |  | X | 4 | 5 | 5 | 5 | 2 | 5 |  |
| Leadership Capacity | Does the president have support from the National Congress? | X |  | X |  |  |  | 3 | 5 | 4 | 1 | 0 | 1 |  |
|  | Does the president have the support of most governors? | X |  | X |  |  |  | 3 | 4 | 4 | 1 | 1 | 1 |  |
|  | Does the governor, regional leader, or mayor support or ideologically align with the president or other government leaders in different spheres? | X |  |  | X | X | X | 2 | 2 | 1 | 2 | 1 | 1 |  |
|  | Does the governor/ regional leader/ mayor have party alignment with the president? | X |  |  | X | X | X | 2 | 2 | 0 | 3 | 2 | 2 |  |
| Comments: | | | | | | | | | | | | | | |

Dimension 3 – Regulation (care coordination across the health system)

| Sub-dimensions | Indicators | Pre-filled (Stages 1 + 2) | | | | | | Filled in by the interviewee | | | | | |  |
| --- | --- | --- | --- | --- | --- | --- | --- | --- | --- | --- | --- | --- | --- | --- |
|  |  | Confirmed and/or proposed | Not confirmed and/or proposed | Fed. | Stat. | Reg. | Mun. | Health System | Health System Resilience | Fed. | Stat. | Reg. | Mun. |  |
| Regulation | Is there a federal/ state/ regulatory/ municipal regulation designed for emergencies and/or epidemics? | X |  | X | X | X | X | 4 | 4 | 4 | 4 | 2 | 4 |  |
|  | Is there a federal regulation for emergencies and/or epidemics regulatory complexes? | X |  | X |  |  |  | 4 | 5 | 5 | 3 | 1 | 1 |  |
| Structure and Healthcare Network | Is there a regulatory complex for managing service delivery? | X |  |  | X | X | X | 4 | 4 | 2 | 4 | 1 | 3 |  |
|  | Is there a regulatory complex for managing both public and private services? | X |  |  | X | X | X | 4 | 4 | 3 | 5 | 3 | 4 |  |
|  | Is there guidance on public and private services operating in a PHE? If so, how is it carried out? | X |  | X | X | X | X | 4 | 3 | 4 | 4 | 2 | 3 |  |
|  | What is the capacity for creating or expanding national/ state/ regional services during a PHE? | X |  | X | X | X | X | 4 | 4 | 4 | 5 | 4 | 5 |  |
|  | How is patient transportation between municipalities/states in the health region coordinated during a PHE? | X |  | X | X | X | X | 2 | 2 | 1 | 3 | 3 | 4 |  |
|  | Is there ongoing, real-time mapping of the hospital inpatient beds and intensive care unit (ICU) occupancy during a PHE? | X |  |  | X | X | X | 4 | 2 | 3 | 5 | 4 | 5 |  |
| Care Management | What is the target and effective coverage ratio in primary, specialized, and urgent/emergency care networks? | X |  |  | X | X | X | 3 | 3 | 2 | 5 | 4 | 4 |  |
|  | What is the ratio between expected and actual production of internal medicine consult services in primary, specialized, and urgent/emergency care networks? | X |  |  | X | X | X | 2 | 3 | 1 | 4 | 2 | 3 |  |
|  | What is the ratio between the target and effective coverage in specialized services? | X |  |  | X | X | X | 2 | 2 | 1 | 2 | 2 | 2 |  |
|  | What is the target and effective coverage ratio for low-, medium-, and high-complexity hospital admissions? | X |  |  | X | X | X | 3 | 3 | 2 | 4 | 4 | 4 |  |
|  | Is there a defined care pathway that outlines patient flows at different levels of the health network during a PHE? If so, how is it carried out? | X |  |  | X | X | X | 4 | 3 | 2 | 5 | 4 | 5 |  |
| Comments: | | | | | | | | | | | | | | |

Dimension 4 – Resources (Financing)

| Sub-dimensions | Indicators | Pre-filled (Stages 1 + 2) | | | | | | Filled in by the interviewee | | | | | |  |
| --- | --- | --- | --- | --- | --- | --- | --- | --- | --- | --- | --- | --- | --- | --- |
|  |  | Confirmed and/or proposed | Not confirmed and/or proposed | Fed. | Stat. | Reg. | Mun. | Health System | Health System Resilience | Fed. | Stat. | Reg. | Mun. |  |
| Financial Resources | Public health expenditure per capita | X |  | X | X | X | X | 3 | 4 | 4 | 4 | 2 | 4 |  |
|  | Percentage of own resources vs. total resources applied in health | X |  | X |  |  |  | 2 | 4 | 4 | 4 | 1 | 4 |  |
|  | Percentage of own resources vs. total resources from intergovernmental transfers applied in health | X |  |  | X |  | X | 3 | 3 | 3 | 4 | 1 | 4 |  |
|  | Expenditure in primary, secondary, and tertiary care, per capita | X |  | X | X | X |  | 4 | 3 | 5 | 5 | 1 | 5 |  |
|  | Expenditure in primary care, per capita | X |  |  |  |  | X | 3 | 3 | 3 | 3 | 0 | 4 |  |
|  | What is the percentage of your resources allocated to health human resources? | X |  | X | X |  | X | 3 | 3 | 2 | 4 | 1 | 4 |  |
|  | What is the percentage of financial resources from legislative amendments allocated to health human resources? | X |  |  | X |  | X | 3 | 2 | 1 | 3 | 1 | 3 |  |
| Management Tools | How can the federal fiscal capacity handle the surge in demand caused by a PHE (planning vs. execution, federal government payment capacity indicator)? | X |  | X |  |  |  | 3 | 3 | 4 | 1 | 1 | 1 |  |
|  | What is the fiscal capacity of the states and municipalities to handle the increased demand caused by a PHE (planning times execution, municipal-level design, and adaptation of pay-for-performance municipal [*indicador da capacidade de pagamento dos municípios (CAPAG)*], and the indicator of the state/federal transfers)? | X |  |  | X |  | X | 3 | 4 | 2 | 5 | 0 | 5 |  |
|  | Is there a dedicated contingency fund for PHE situations? | X |  | X | X |  | X | 3 | 3 | 5 | 3 | 0 | 3 |  |
|  | Is there a shared contingency fund for PHE situations? | X |  |  |  | X |  | 2 | 2 | 4 | 2 | 1 | 1 |  |
|  | Is there an agreement with the private sector for a financing partnership in case of a PHE? |  | X | X | X | X | X | 2 | 1 | 3 | 2 | 0 | 2 |  |
| Comments: | | | | | | | | | | | | | | |

Dimension 5 – Resources (Health Workforce)

| Sub-dimensions | Indicators | Pre-filled (Stages 1 + 2) | | | | | | Filled in by the interviewee | | | | | |  |
| --- | --- | --- | --- | --- | --- | --- | --- | --- | --- | --- | --- | --- | --- | --- |
|  |  | Confirmed and/or proposed | Not confirmed and/or proposed | Fed. | Stat. | Reg. | Mun. | Health System | Health System Resilience | Fed. | Stat. | Reg. | Mun. |  |
| Distribution of Professionals | Number of physicians per 10,000 inhabitants | X |  | X | X | X | X | 4 | 4 | 4 | 4 | 2 | 4 |  |
|  | Number of nurses per 10,000 inhabitants | X |  | X | X | X | X | 4 | 4 | 4 | 5 | 2 | 4 |  |
|  | Number of nursing technicians per 10,000 inhabitants | X |  | X | X | X | X | 4 | 3 | 3 | 4 | 3 | 5 |  |
|  | Number of physical therapists per 10,000 inhabitants | X |  | X | X | X | X | 4 | 3 | 4 | 4 | 3 | 4 |  |
|  | Number of Family Health Strategy [*Estratégia Saúde da Família (ESF)]* and Primary Care [(AB)] teams per 10,000 inhabitants | X |  | X | X | X | X | 5 | 3 | 2 | 2 | 3 | 5 |  |
| Management Tools | Are there alternative ways to manage the health workforce (e.g., social health organizations, foundations, etc.)? | X |  | X | X |  | X | 3 | 1 | 1 | 3 | 0 | 3 |  |
|  | Percentage of health professionals hired under direct vs. indirect administration | X |  | X | X | X | X | 3 | 1 | 1 | 2 | 1 | 2 |  |
|  | Are there health professionals specialized in services provided via telecommunications (telemedicine)? | X |  | X | X |  | X | 4 | 3 | 2 | 4 | 2 | 4 |  |
|  | Is there a support structure for health professionals due to work overload, emotional stress (burnout), and high rates of PHE? |  | X | X | X |  | X | 3 | 3 | 2 | 3 | 1 | 3 |  |
|  | Is there training for health professionals to provide care safely and with personal protection in a PHE? | X |  | X | X |  | X | 5 | 3 | 4 | 4 | 1 | 3 |  |
|  | Is there a process for identifying and recruiting additional health professionals to replace absent staff in care teams during a PHE? |  | X | X | X |  | X | 3 | 3 | 3 | 5 | 1 | 5 |  |
|  | Is there a coordination structure for engaging volunteers in non-technical activities (e.g., transporting personnel and family members) during a PHE? |  | X | X | X |  | X | 3 | 2 | 3 | 3 | 0 | 4 |  |
|  | Is there an agreement with the private sector to partner on human resources in case of a PHE? |  | X | X | X | X | X | 3 | 3 | 3 | 4 | 2 | 4 |  |
| Comments: | | | | | | | | | | | | | | |

Dimension 6 – Resources (Physical Resources)

| Sub-dimensions | Indicators | Pre-filled (Stages 1 + 2) | | | | | | Filled in by the interviewee | | | | | |  |
| --- | --- | --- | --- | --- | --- | --- | --- | --- | --- | --- | --- | --- | --- | --- |
|  |  | Confirmed and/or proposed | Not confirmed and/or proposed | Fed. | Stat. | Reg. | Mun. | Health System | Health System Resilience | Fed. | Stat. | Reg. | Mun. |  |
| Management Tools | Is there a structure to coordinate appropriate cleaning supplies/services in a PHE? |  | X | X | X |  | X | 3 | 3 | 2 | 3 | 3 | 3 |  |
|  | Is there a sufficient stock of personal protective equipment (PPE) for a PHE? |  | X | X | X |  | X | 2 | 4 | 5 | 4 | 3 | 4 |  |
|  | Is there a structure for managing and maintaining critical physical resources/equipment during a PHE? | X |  | X | X | X | X | 3 | 3 | 3 | 4 | 4 | 4 |  |
|  | Is there a structure to manage patient transportation in a PHE? | X |  | X | X | X | X | 3 | 4 | 4 | 4 | 4 | 4 |  |
|  | Is there an agreement with the private sector for a partnership on physical resources in case of a PHE? |  | X | X | X | X | X | 3 | 3 | 3 | 2 | 1 | 2 |  |
| Care Network | Is there a network of emergency care that includes critical care units [Unidade de Pronto Atendimento (UPAs)] (e.g., intensive care units or high dependency units) and the Mobile Emergency Care Service (MECs) [ Serviço de Atendimento Móvel de Urgência (SAMU)]? | X |  | X | X |  | X | 5 | 3 | 3 | 4 | 4 | 4 |  |
|  | Number of primary care facilities per 4,000,000 inhabitants |  | X | X | X | X | X | 3 | 2 | 1 | 2 | 2 | 1 |  |
|  | Number of secondary care facilities per 4,000,000 inhabitants |  | X | X | X | X |  | 4 | 2 | 2 | 3 | 3 | 2 |  |
|  | Number of tertiary care facilities per 4,000,000 inhabitants |  | X | X | X | X |  | 4 | 2 | 3 | 3 | 2 | 1 |  |
| Infrastructure | Number of hospital inpatient beds per 10,000 inhabitants | X |  | X | X | X |  | 5 | 5 | 5 | 4 | 4 | 4 |  |
|  | Number of urgent and emergency care beds per 10,000 inhabitants | X |  | X | X | X |  | 5 | 4 | 4 | 4 | 4 | 4 |  |
|  | Number of ventilators/respiratory protection per 10,000 inhabitants |  | X | X | X | X |  | 2 | 4 | 3 | 3 | 3 | 2 |  |
|  | Number of recovery/observation beds per 10,000 inhabitants | X |  | X | X | X | X | 3 | 2 | 1 | 3 | 3 | 3 |  |
|  | Number of equipment (depending on the type of PHE) per 400,000 inhabitants | X |  | X | X | X | X | 3 | 2 | 2 | 3 | 3 | 2 |  |
| Comments: | | | | | | | | | | | | | | |

Dimension 7 – Medicines

| Sub-dimensions | Indicators | Pre-filled (Stages 1 + 2) | | | | | | Filled in by the interviewee | | | | | |  |
| --- | --- | --- | --- | --- | --- | --- | --- | --- | --- | --- | --- | --- | --- | --- |
|  |  | Confirmed and/or proposed | Not confirmed and/or proposed | Fed. | Stat. | Reg. | Mun. | Health System | Health System Resilience | Fed. | Stat. | Reg. | Mun. |  |
| Management Tools | Is there an administrative structure to manage the essential medication stock for the health system? | X |  | X | X |  | X | 5 | 2 | 4 | 4 | 2 | 4 |  |
|  | Are there drug manufacturing partnerships with other governmental entities or organizations? | X |  | X | X | X | X | 4 | 4 | 4 | 2 | 1 | 1 |  |
|  | Are there specific programs or policies to distribute medicine to the population? | X |  | X | X | X | X | 5 | 3 | 4 | 4 | 3 | 4 |  |
|  | Are there specific programs or policies for developing new medications? | X |  | X | X |  |  | 5 | 5 | 5 | 2 | 0 | 0 |  |
| Regulatory Market | Average approval/registration time for new medicines | X |  | X |  |  |  | 4 | 2 | 3 | 0 | 0 | 0 |  |
|  | Average number of new products approved/registered (entry) | X |  | X |  |  |  | 4 | 1 | 3 | 0 | 0 | 0 |  |
|  | Steps/processes required for the entry of new medicines | X |  | X |  |  |  | 4 | 1 | 3 | 0 | 0 | 0 |  |
|  | Types of medicines entering the country (generics, etc.) | X |  | X |  |  |  | 4 | 1 | 3 | 0 | 0 | 0 |  |
|  | Public procurement indicator for medicines | X |  | X | X |  | X | 4 | 1 | 4 | 2 | 0 | 2 |  |
|  | Indicator of reported shortages | X |  | X | X |  | X | 5 | 2 | 4 | 4 | 2 | 4 |  |
|  | Indicator of medicine distribution | X |  | X | X |  | X | 3 | 1 | 2 | 2 | 1 | 2 |  |
| Pharmaceutical Services | Is there a specialized structure for managing pharmaceutical services? | X |  | X | X |  | X | 4 | 3 | 4 | 3 | 1 | 3 |  |
|  | Is there a system for monitoring inventory, planning, expiration, classification, and human resources in pharmaceutical services? |  | X | X | X |  | X | 5 | 3 | 4 | 4 | 1 | 4 |  |
| Comments: | | | | | | | | | | | | | | |

Dimension 8 – Technology

| Sub-dimensions | Indicators | Pre-filled (Stages 1 + 2) | | | | | | Filled in by the interviewee | | | | | |  |
| --- | --- | --- | --- | --- | --- | --- | --- | --- | --- | --- | --- | --- | --- | --- |
|  |  | Confirmed and/or proposed | Not confirmed and/or proposed | Fed. | Stat. | Reg. | Mun. | Health System | Health System Resilience | Fed. | Stat. | Reg. | Mun. |  |
| Information Systems | Is there a structured system for systematic data collection, storage, and analysis to monitor the population’s health? | X |  | X | X |  | X | 4 | 3 | 4 | 3 | 0 | 3 |  |
|  | Is there any system to manage the supply chain in healthcare? | X |  | X | X |  | X | 4 | 2 | 4 | 3 | 0 | 3 |  |
|  | Is there any system to monitor potential external shocks, emergencies, epidemics, or pandemics? |  | X | X | X |  | X | 3 | 3 | 3 | 1 | 0 | 1 |  |
|  | Is there a structure for expanding information and communication technologies during a PHE? | X |  | X | X |  | X | 4 | 2 | 3 | 3 | 1 | 3 |  |
|  | Is any system automatically applying data science to inform actions in the health sector? |  | X | X | X |  | X | 3 | 2 | 2 | 2 | 0 | 1 |  |
|  | Is there technology that provides services via telecommunications (telemedicine)? | X |  | X | X |  | X | 4 | 3 | 4 | 3 | 1 | 3 |  |
|  | Is there the capacity to achieve interoperability across different information systems quickly? | X |  | X | X |  | X | 3 | 2 | 2 | 2 | 0 | 1 |  |
| Communication and Support | Is there a capacity to generate information with practical value for decision-making? | X |  | X | X |  | X | 4 | 3 | 3 | 3 | 1 | 3 |  |
|  | Is there a support structure for municipalities in implementing information systems (IS)? | X |  | X | X |  |  | 3 | 2 | 3 | 2 | 1 | 0 |  |
|  | Is there any health communication system for the population? | X |  | X | X |  | X | 4 | 3 | 4 | 3 | 1 | 3 |  |
| Comments: | | | | | | | | | | | | | | |

Dimension 9 – Service Delivery

| Sub-dimensions | Indicators | Pre-filled (Stages 1 + 2) | | | | | | Filled in by the interviewee | | | | | |  |
| --- | --- | --- | --- | --- | --- | --- | --- | --- | --- | --- | --- | --- | --- | --- |
|  |  | Confirmed and/or proposed | Not confirmed and/or proposed | Fed. | Stat. | Reg. | Mun. | Health System | Health System Resilience | Fed. | Stat. | Reg. | Mun. |  |
| Public Health | What is the percentage of the population covered by the Family Health Strategy (FHS) [*Estratégia Saúde da Família*]? | X |  | X | X | X | X | 5 | 2 | 2 | 3 | 1 | 5 |  |
|  | What is the percentage of the population covered by primary care teams? | X |  | X | X | X | X | 4 | 2 | 2 | 3 | 1 | 4 |  |
|  | What is the percentage of population coverage for influenza vaccination? | X |  | X | X | X | X | 4 | 2 | 3 | 3 | 2 | 4 |  |
|  | What is the percentage of population coverage for COVID-19 vaccination? | X |  | X | X | X | X | 4 | 3 | 3 | 3 | 2 | 4 |  |
|  | What is the percentage of children receiving tetra/penta/hexavalent vaccines? |  | X | X | X | X | X | 4 | 3 | 3 | 3 | 2 | 4 |  |
| Primary Care | What is the percentage of live births with fewer than seven prenatal visits? | X |  | X | X | X | X | 5 | 1 | 3 | 4 | 4 | 5 |  |
|  | Rate of vaginal deliveries performed |  | X | X | X | X | X | 4 | 1 | 3 | 3 | 3 | 4 |  |
|  | Infant mortality rate | X |  | X | X | X | X | 4 | 2 | 3 | 3 | 3 | 4 |  |
|  | Maternal mortality rate | X |  | X | X | X | X | 4 | 2 | 3 | 3 | 3 | 4 |  |
| Specialized Care | General estimated mortality rate (by facility type and specialty) | X |  | X | X |  | X | 4 | 1 | 4 | 3 | 3 | 3 |  |
|  | Bed occupancy rate and turnover (by facility type and specialty) | X |  | X | X |  | X | 4 | 3 | 3 | 5 | 4 | 5 |  |
|  | Number of hospital admissions (by facility type and specialty) | X |  | X | X |  | X | 3 | 3 | 2 | 3 | 2 | 4 |  |
|  | Average time to conduct tests and receive results | X |  | X | X |  | X | 4 | 3 | 3 | 5 | 4 | 5 |  |
| Comments: | | | | | | | | | | | | | | |

General Comments – Full Framework

| Comments: |
| --- |
