# Supplementary material for: Building a health system resilience framework: national, state, regional, and local perspectives
Source: Lancet Reg Health Am. 2025 Dec 11;54:101334. doi: 10.1016/j.lana.2025.101334 (PMC12757546; doi:10.1016/j.lana.2025.101334)
Supplement: Appendix 4 [file mmc4.docx]

Appendix 4: COREQ checklist

Consolidated criteria for reporting qualitative studies (COREQ): 32-item checklist

Developed from:

Tong A, Sainsbury P, Craig J. Consolidated criteria for reporting qualitative research (COREQ): a 32-item checklist for interviews and focus groups. International Journal for Quality in Health Care. 2007. Volume 19, Number 6: pp. 349 – 357

| **Item No** | | **Guide Questions/Description** | **Reported on Page #** |  |
| --- | --- | --- | --- | --- |
| **Domain 1: Research team and reflexivity** | | | |  |
| **Personal Characteristics** | | | |  |
| 1. Interviewer/ facilitator | | Which author/s conducted the interview or focus group? | The first author conducted the interviews (we preferred not to insert in the text). There was no focus group. |  |
| 2. Credentials | | What were the researcher’s credentials? E.g., PhD, MD | The research team is formed by PhDs and one MD (we preferred not to insert in the text). |  |
| 3. Occupation | | What was their occupation at the time of the study? | Four authors are assistant or associate professors, and one author is a researcher (we preferred not to insert in the text). |  |
| 4. Gender | | Was the researcher male or female? | One female and four male researchers, where the one male researcher executed the interviews (page 4). |  |
| 5. Experience and training | | What experience or training did the researcher have? | All the authors have experience in interviews and health system resilience (we preferred not to insert in the text). |  |
| **Relationship with participants** | | | |  |
| 6. Relationship established | | Was a relationship established prior to study commencement? | No. “The invitation was sent through the formal email contacts from the official websites” (page 4). |  |
| 7. Participant knowledge of the interviewer | | What did the participants know about the researcher? e.g. personal goals, reasons for doing the research? | “The invitation, with the project goals and researcher affiliations, was sent through the formal email contacts from the official websites.” (page 4). |  |
| 8. Interviewer characteristics | | What characteristics were reported about the interviewer/facilitator? e.g. Bias, assumptions, reasons and interests in the research topic | “The invitation, with the project goals and researcher affiliations, was sent through the formal email contacts from the official websites.” (page 4). |  |
| **Domain 2: study design** | | |  |  |
| **Theoretical framework** | | |  |  |
| 9. Methodological orientation and Theory | What methodological orientation was stated to underpin the study? e.g. grounded theory, discourse analysis, ethnography, phenomenology, content analysis | “We applied a qualitative deductive-inductive approach divided into three phases: phase 1 - create the HSR dimensions from an international perspective; phase 2 - adapt the dimensions to the Brazilian health system and establish the indicators of each dimension; and phase 3 - validate the Brazilian HSR framework through experts' agreement. We choose the deductive-inductive approach to (a) identify already existent health system resilience dimensions, subdimensions and indicators – deductive; and (b) discover new dimensions and indicators from the health system resilience experts – inductive (page 4). |  |  |
| **Participant selection** | | |  |  |
| 10. Sampling | How were participants selected? e.g., purposive, convenience, consecutive, snowball | “The experts were selected purposively, based on their academic and/or practical knowledge and expertise in the health system resilience dimensions and indicators. The experts were contacted by email, signed a consent form and the interviews were realized and recorded through the zoom platform.  The reporting process followed the COREQ elements (team/reflexivity; study design; analysis), available in Appendix 4. We used triangulation across three phases, prespecified codebooks, dual-reviewed coding in Phase 2 subsamples, and audit trails. A Delphi was considered; we prioritised semi-structured interviews to elicit context-specific insights across governance levels and then used a structured validation interview (Phase 3) to confirm indicator inclusion and level allocation. Interview guide and codebook are provided in Appendix 1–2.” (page 6) |  |  |
| 11. Method of approach | How were participants approached? e.g., face-to-face, telephone, mail, email | “The experts were selected purposively, based on their academic and/or practical knowledge and expertise in the health system resilience dimensions and indicators. The experts were contacted by email, signed a consent form and the interviews were realized and recorded through the zoom platform.  The reporting process followed the COREQ elements (team/reflexivity; study design; analysis), available in Appendix 4. We used triangulation across three phases, prespecified codebooks, dual-reviewed coding in Phase 2 subsamples, and audit trails. A Delphi was considered; we prioritised semi-structured interviews to elicit context-specific insights across governance levels and then used a structured validation interview (Phase 3) to confirm indicator inclusion and level allocation. Interview guide and codebook are provided in Appendix 1–2.” (page 6) |  |  |
| 12. Sample size | How many participants were in the study? | “Using a unique set of 48 international and national health system experts and capturing the country’s specific challenges and strengths, our framework can inform targeted policy interventions, ultimately contributing to the HSR literature and designing more resilient and equitable health systems.” (page 4)  “The data collection and analysis followed the three phases represented in Figure 1. Phase 1 is dedicated to foundational planning and framework development. It focuses on identifying key dimensions of health system resilience, drawing insights from literature reviews (appendix 1) and then crossing with the 21 experts' interviews. The phase 1 interviews were anchored in WHO Building Blocks to ensure comprehensiveness and comparability with established performance domains; we then iteratively translated inputs into resilience-specific subdimensions and indicators, separating steady-state performance from adaptive capacities. Initial drafts of dimensions are crafted during this phase by a deductive-inductive approach, forming ten dimensions, which are the backbone of the framework.  FIGURE 1 HERE  Phase 2 amplifies the initial framework by testing the dimensions and developing the indicators to represent each. Based on phase 1 and the new literature review to collect more indicators for each one of the dimensions, 22 Brazilian experts were interviewed (appendix 2), generating through deductive-inductive analysis the Brazilian HSR framework proposal. Finally, the final phase validates the framework by interviewing five experts and defining the government and geographical level of the indicators using a deductive approach.” (pages 5-6) |  |  |
| 13. Non-participation Setting | How many people refused to participate or dropped out? Reasons? | 6 experts refused because of no agenda to schedule the interview (we preferred not to insert in the text). |  |  |
| 14. Setting of data collection | Where was the data collected? e.g., home, clinic, workplace | Zoom platform (page 6). |  |  |
| 15. Presence of nonparticipants | Was anyone else present besides the participants and researchers? | N/A |  |  |
| 16. Description of sample | What are the important characteristics of the sample? e.g. demographic data, date | “Using a unique set of 48 international and national health system experts and capturing the country’s specific challenges and strengths, our framework can inform targeted policy interventions, ultimately contributing to the HSR literature and designing more resilient and equitable health systems.” (page 4).  “The experts were selected purposively, based on their academic and/or practical knowledge and expertise in the health system resilience dimensions and indicators.” (page 6). |  |  |
| **Data collection** | | |  | No |
| 17. Interview guide | Were questions, prompts, and guides provided by the authors? Was it pilot tested? | “The experts were selected purposively, based on their academic and/or practical knowledge and expertise in the health system resilience dimensions and indicators. The experts were contacted by email, signed a consent form and the interviews were realized and recorded through the zoom platform.  The reporting process followed the COREQ elements (team/reflexivity; study design; analysis) available in Appendix 4. We used triangulation across three phases, prespecified codebooks, dual-reviewed coding in phase 2 subsamples, and audit trails. A Delphi was considered, but we prioritised semi-structured interviews to elicit context-specific insights across governance levels and then used a structured validation interview (Phase 3) to confirm indicator inclusion and level allocation. Interview guide and codebook are provided in Appendix 1–2.” (page 6). |  |  |
| 18. Repeat interviews | Were repeat interviews carried out? If yes, how many? | N/A |  |  |
| 19. Audio/visual recording | Did the research use audio or visual recording to collect the data? | “The experts were contacted by email, signed a consent form and the interviews were realized and recorded through the zoom platform.” (page 6). |  |  |
| 20. Field notes | Were field notes made during and/or after the interview or focus group? | N/A |  |  |
| 21. Duration | What was the duration of the interviews or focus group? | “All interviews of the three phases happened between November 5th, 2021, and September 19th, 2023, with an average duration of 49 minutes.” (page 6). |  |  |
| 22. Data saturation | Was data saturation discussed? | “Phases 1 and 2 of our qualitative deductive-inductive approach generated a Brazilian HSR framework proposal with 9 dimensions and 112 indicators (appendix 3). The final framework has nine health system resilience dimensions, 18 subdimensions and 65 indicators validated at national, state, regional and local levels (Table 2).” (page 6). |  |  |
| 23. Transcripts returned | Were transcripts returned to participants for comment and/or correction? | N/A |  |  |
| **Domain 3: analysis and findings** | | |  |  |
| **Data analysis** | | |  |  |
| 24. Number of data coders | How many data coders coded the data? | 1 (we preferred not to insert in the text). |  |  |
| 25. Description of the coding tree | Did the authors provide a description of the coding tree? | N/A |  |  |
| 26. Derivation of themes | Were themes identified in advance or derived from the data? | “Phases 1 and 2 of our qualitative deductive-inductive approach generated a Brazilian HSR framework proposal with 9 dimensions and 112 indicators (appendix 3). The final framework has nine health system resilience dimensions, 18 subdimensions and 65 indicators validated at national, state, regional and local levels (Table 2).  Table 3 presents the final HSR framework and all the details of the development process. The table shows the results from stages 1 and 2 (grey area) and the sum of the experts' answers from stage 3. To be part of the final HSR framework, the indicator should achieve the criterion of having at least 3 answers in the “Health System Resilience Column”. Also, the same criterion was applied to the “Health System” and “government levels (federal, state, regional and municipal)” columns.” (pages 6-7). |  |  |
| 27. Software | What software, if applicable, was used to manage the data? | N/A |  |  |
| 28. Participant checking | Did participants provide feedback on the findings? | N/A |  |  |
| **Reporting** | | |  |  |
| 29. Quotations presented | Were participant quotations presented to illustrate the themes/findings? Was each quotation identified? e.g., participant number | We did not use quotations to illustrate the main findings. |  |  |
| 30. Data and findings consistent | Was there consistency between the data presented and the findings? | Yes. (pages 4-12). |  |  |
| 31. Clarity of major themes | Were major themes clearly presented in the findings? | Yes (pages 6-11). |  |  |
| 32. Clarity of minor themes | Is there a description of diverse cases or a discussion of minor themes? | N/A |  |  |
