# Supplementary material for: Building a health system resilience framework: national, state, regional, and local perspectives
Source: Lancet Reg Health Am. 2025 Dec 11;54:101334. doi: 10.1016/j.lana.2025.101334 (PMC12757546; doi:10.1016/j.lana.2025.101334)
Supplement: Resumen ESP [file mmc5.pdf]

__Editorial disclaimer:__ This translation in Spanish was submitted by the authors and we reproduce it as supplied. It has not been peer reviewed. Our editorial processes have only been applied to the original abstract in English, which should serve as reference for this manuscript.

Resumen

La resiliencia de los sistemas de salud (HSR) es esencial para mantener las funciones esenciales de forma equitativa frente a estresores agudos y crónicos en sistemas descentralizados. Desarrollamos y validamos un framework de HSR adaptado al contexto de Brasil que distingue el desempeño en situación estable de las capacidades específicas de resiliencia y asigna responsabilidades entre los niveles federal, estatal, regional y municipal. Mediante un enfoque cualitativo deductivo–inductivo en tres fases, con la participación de 48 expertos internacionales y nacionales, identificamos nueve dimensiones, 18 subdimensiones y 65 indicadores que priorizan la coherencia de la gobernanza, las estrategias de refuerzo de la fuerza laboral, la regulación en situaciones de emergencia, el monitoreo en tiempo real y el acceso a tecnologías críticas. El framework aclara los límites entre el desempeño general del sistema de salud y las funciones adaptativas, de absorción y transformadoras, y especifica cómo los gestores pueden aplicarlo en la práctica mediante pasos estructurados de definición de alcance, mapeo, puntuación, priorización, planificación y monitoreo. Aunque fue diseñado para el Sistema Único de Salud (SUS) de Brasil, la lógica de desarrollo se generaliza a otros contextos descentralizados con la adecuada reasignación de responsabilidades y calibración a las normas nacionales de financiamiento. Esta herramienta orientada a las políticas respalda el fortalecimiento operativo de la resiliencia en sistemas complejos y multinivel.

Palabras clave: Resiliencia del sistema de salud; Descentralización; Sistema de salud; Sistema Único de Salud; Gobernanza.
