# Supplementary material for: Building a health system resilience framework: national, state, regional, and local perspectives
Source: Lancet Reg Health Am. 2025 Dec 11;54:101334. doi: 10.1016/j.lana.2025.101334 (PMC12757546; doi:10.1016/j.lana.2025.101334)
Supplement: Resumo POR [file mmc6.pdf]

Editorial disclaimer: *This translation in Portuguese was submitted by the authors and we reproduce it as supplied. It has not been peer reviewed. Our editorial processes have only been applied to the original abstract in English, which should serve as reference for this manuscript.*

Resumo

A resiliência dos sistemas de saúde (HSR) é essencial para manter funções essenciais de forma equitativa diante de estressores agudos e crônicos em sistemas descentralizados. Desenvolvemos e validamos um framework de HSR adaptado ao contexto brasileiro que distingue o desempenho em situação de normalidade das capacidades específicas de resiliência e atribui responsabilidades entre os níveis federal, estadual, regional e municipal. Utilizando uma abordagem qualitativa dedutivo–indutiva em três fases, com 48 especialistas internacionais e nacionais, identificamos nove dimensões, 18 subdimensões e 65 indicadores que priorizam a coerência da governança, estratégias de ampliação da força de trabalho, regulação em situações de emergência, monitoramento em tempo real e acesso a tecnologias críticas. O framework esclarece os limites entre o desempenho geral do sistema de saúde e as funções adaptativas, de absorção e transformadoras, e especifica como gestores podem aplicá-lo na prática por meio de etapas estruturadas de definição de escopo, mapeamento, pontuação, priorização, planejamento e monitoramento. Embora tenha sido desenvolvido para o Sistema Único de Saúde (SUS) do Brasil, a lógica de desenvolvimento se generaliza para outros contextos descentralizados, com adequada realocação de responsabilidades e calibração às regras nacionais de financiamento. Essa ferramenta voltada para políticas públicas apoia o fortalecimento operacional da resiliência em sistemas complexos e multiníveis.

Palavras-chave: Resiliência do sistema de saúde; Descentralização; Sistema de saúde; Sistema Único de Saúde; Governança.
